# Three-Dimensional Printing of Hydrogel as Skin Substitute and Comparative Evaluation of Melanin Production

**DOI:** 10.3390/bioengineering12030270

**Published:** 2025-03-09

**Authors:** Mohammad Zafaryab, Komal Vig

**Affiliations:** Department of Biological Sciences, Alabama State University, Montgomery, AL 36104, USA; mzafaryab@alasu.edu

**Keywords:** hydrogel, scaffold, tissue engineering, viability, fluorescence microscopy

## Abstract

Cell culture in two dimensions has been the main instrument in cellular and molecular biology. But there are limitations to two-dimensional culture when it comes to tissue engineering and in vivo reproduction. Tissue engineering technology enabled the creation of biomedical scaffolds, which are mostly utilized to biofabricate different artificial human organs. Tissue architecture that encourage cell proliferation can be produced using direct bioprinting technology. The development of bioinks for 3D bioprinting is consistently seen as a problem in the domains of biofabrication and tissue engineering. This study aimed to determine if Fibroblasts and Keratinocytes could grow on hydrogel scaffolds as efficiently as they can in the culture plates. Melanocytes were co-cultured, and the production of melanin was assessed in a two- and three-dimensional culture system. Scaffolds were fabricated using 8% alginate and 6% gelatin and 3D-printed using a cell link printer. FTIR was used to determine the precise composition of the gels. SEM analysis was performed for the cells present in gel and the topology of the cells. In addition, 8% alginate and 6% alginate gel scaffolds were analyzed for swelling and degradation over time in the cell growth medium and PBS. Furthermore, a gene expression study of cell cultures on scaffolds was performed through qPCR. A live/dead assay was performed to determine cell viability for cells grown on scaffolds for 7, 14, and 21 days. Most of the cells were shown to be viable, similar to the control cells grown on a plate. The findings from the SEM showed that cells were grown on the gel surface, remained viable even after 21 days, and displayed circular cells stacked three-dimensionally on the gel surface in the 3D scaffold. The MTT assay was performed to check the viability of cells cultured on a 3D-printed scaffold for 1, 5, and 15 days. We observed about 40% viable cells after 15 days, as shown by the MTT assay. Furthermore, a co-culture study with Melanocyte showed an increased production of melanin in a 3D culture as compared to a 2D culture. Our findings suggest that an alginate and gelatin polymer can be used as a cellular matrix for epithelial cell culture. Further, in vivo and ex vivo experiments are needed to validate the results for future applications in tissue engineering for wound healing and other tissue engineering domains.

## 1. Background

The skin is the biggest and most susceptible organ. Burns are challenging to treat with modern technology because they need skin grafts to promote healing. Skin transplants exacerbate problems and cause more wounds. Wounds that do not heal or heavy scar tissue might result from improper healing. Additionally, scars may become elevated or turn deep pink or red. These problems are more likely to occur in deeper cuts [1]. The dermis and epidermis are the two layers that make up the skin. Fibroblasts reside in the dermis, whereas Keratinocytes inhabit the epidermis, the topmost layer. Chronic diseases can slow down the healing of wounds and raise the risk of infections and consequences. Scar tissue can be less noticeable with the use of steroids, wound dressings, and scar revision surgery [2]. Wound healing is a highly regulated and precise biological process that involves phases of hemostasis, inflammation, proliferation, and remodeling. The currently available methods for wound healing include the application of herbal and natural products, antimicrobial agents, and healing boosters. There are several issues with the technologies currently employed to promote wound healing. The attachment of skin cells to a scaffold to provide structure is the subject of recent research on methods to enhance wound healing. Utilizing a patient’s own skin cells helps speed up the healing process, which lowers the likelihood of problems and the extent of scarring [3]. Advancements in technology have enabled the transition from two-dimensional to three-dimensional cell growth [4].

Three-dimensional (3D) printing is a cutting-edge technique that could revolutionize tissue engineering and regeneration. This technique offers the chance to precisely deposit materials loaded with various cells in a layer-by-layer fashion to produce intricate tissue-engineered structures that closely resemble the intricacy of the original tissue [5,6,7,8]. Three-dimensional bioprinting technologies can be grouped into three categories, extrusion-based, inkjet-based, and laser-assisted bioprinting, depending on the operating principle. Extrusion-based bioprinting is currently considered the most promising method for creating skin or soft tissue structures. Hydrogels are used in this technology as both building materials and cell delivery vehicles due to their unique physical, chemical, and biological properties. They mimic the hygroscopic nature of the extracellular matrix and provide an ideal environment for cell growth and differentiation [9,10]. The ideal hydrogels for 3D bioprinting must meet three fundamental requirements: (a) high cell viability through biocompatibility, (b) proper printing rheology, and (c) rapid crosslinking for 3D structure retention [11]. Natural hydrogels, such hyaluronic acid [12], chitosan [13], collagen [14], and alginate [15], are often employed for 3D bioprinting due to their high biocompatibility. However, bioprinted constructions often lack shape fidelity and collapse due to low viscosity or delayed crosslinking. To circumvent the current constraints, composite hydrogels are frequently used. Several research studies have used 3D-printed alginate/gelatin composite hydrogels for tissue engineering applications, such as an aortic valve conduit [16], bone [17,18], and eye cornea [19]. While alginate hydrogels exhibit viscous behavior at various concentrations, gelatin hydrogels exhibit excellent elastic properties. Alginate and gelatin mixtures have been shown to be good for 3D bioprinting due to their synergistic effect [11]. Another disadvantage with using alginate alone includes that it lacks attachment sites for cells, limiting cell adherence and function. Adding cell-recognition peptides (e.g., RGD peptides) or other biomaterials to alginate hydrogel can boost cell adherence [20,21]. Gelatin is a naturally occurring biopolymer formed from collagen that incorporates patterns such as RGD sequences, hence improving cellular activity. Gelatin, a biopolymer, is an ideal choice for hydrogel printing due to its ease of production and manipulation [22]. In regenerative medicine, scaffolds are crucial because they provide the cells with a cellular matrix to develop in areas where the transplant will resemble natural skin the most [23]. The challenge is in developing a cellular matrix that is affordable, promotes the growth of healthy cells, breaks down over time, and can withstand handling [24].

The purpose of this work was to ascertain whether skin cells and an 8% alginate/6% gelatin scaffold could be used together in skin tissue engineering. Additionally, we aimed to determine whether a gel containing 8% alginate/6% gelatin gel will have improved printability with Fibroblast and HaCaT cell proliferation. Furthermore, a comparative evaluation study has been performed to check the production of melanin in 2D culture and 3D culture, i.e., alginate/gelatin 3D-printed scaffold.

## 2. Materials and Methods

Medium-viscosity alginic acid sodium salt from brown algae was obtained from Acros Organic (CAS No: 9005-38-3). Type B gelatin was obtained from Fisher chemical. Dulbecco’s Modified Eagle Medium (DMEM) and Fetal Bovine Serum (FBS) were purchased from Gibco, and dermal media were purchased from ATCC (USA). Fibroblast, Keratinocyte (HaCaT), and Melanocyte cells were procured from ATCC (USA). The live/dead assay kit was obtained from Thermo Fisher Scientific (Waltham, MA, USA). Basic lab analytic-grade reagents were obtained from Sigma and Merck.

### 2.1. Cell Culture and Maintenance

Fibroblasts and Keratinocytes were cultured in DMEM containing 10% FBS in a tissue culture-graded flask at 37 °C in the presence of 5% CO_2_ in a CO_2_ incubator. After reaching 70% confluency, cells were trypsinized using trypsin and centrifuged at 2500 rpm for 3 min to pellet down the cells. Cells were counted with the countess slide by mixing 10 μL of cells with 10 μL of trypan blue dye using Invitrogen Countess 3 (USA). Melanocyte cells were cultured in melanocyte dermal media containing growth factors under similar culture conditions.

### 2.2. Alginate/Gelatin

We selected 8% (*w*/*v*) alginate and 6% (*w*/*v*) gelatin in a 1:1 ratio for the 3D-printed scaffold used in this study, which showed (a) good pore size, (b) a well-defined border, (c) good resolution, (d) printing accuracy, and (e) overall gel strength. For the experiments, we initially tried different percentages and ratios of alginate and gelatin for 3D printing of the scaffolds. Based on our analysis, we decided upon 8% alginate/6% gelatin, which fulfilled our criteria of pore size, etc. Hydrogel ink was prepared by mixing the 8% *w*/*v* alginate and 6% *w*/*v* gelatin. To make 60 mL of 8% alginate/6% gelatin gel/ink, each of them were dissolved in 30 mL of PBS pH 7.4. To make 60 mL of 8% alginate/6% gelatin gel, each of them were dissolved in 30 mL of hot PBS (pH 7.4). For 8% alginate, 2.4 g of sodium alginate was added in PBS and stirred on a hot plate for 1 hr at 80 °C. Similarly, for 6% gelatin, 1.8 g of gelatin powder was added to pre-hot PBS and stirred for 30 min. Further, prepared 8% alginate and 6% gelatin were mixed in a 1:1 ratio and stirred at 60 °C for 1 hr. The gel was centrifuged at 3000 rpm for 3 min to eliminate any bubbles before putting it into 3 mL pneumatic syringes for 3D printing.

### 2.3. FTIR Analysis

Sodium alginate powder, gelatin powder, and 8% alginate/6% gelatin hydrogel were analyzed using FTIR to detect the presence of characteristic functional groups. The FTIR spectra were scanned at room temperature using the Nicolet™ iS50 FTIR Spectrometer Thermo-fisher scientific (Waltham, MA, USA) with an ATR objective (iS50 ATR). The scanning range used was 650–4000 cm^−1^. The sample platform of the FTIR machine was cleaned with DI water between test samples. Data were collected and analyzed for characteristic peaks.

### 2.4. Printing of Scaffold

The Cellink Bio X Bioprinter was used to print scaffolds. Before every use, the printer was cleaned using a fan and UV light. Under the Bioprinter option, BioModels were selected, and the desired size and dimension were chosen. The scaffolds were printed at 20 × 20 × 1 mm and 30 × 30 × 1 mm sizes for this study. A 100 cm^2^ cleaned glass Petri dish was used as the surface for printing. Under the printer tab, external pressure was turned on. The printhead tool, nozzle size, temperature, pressure, and speed were selected. The 22 G nozzle was used with the 3 mL pneumatic syringe. Further, the infill density, infill percentage, and infill pattern were selected. Once all the settings were set, the printer then manually calibrated to where the nozzle just touched the print surface and started once the bottom was on. Once the scaffold finished printing, it was covered with 2% CaCl_2_ to crosslink the gel polymer for 5 min. The printing parameter was chosen, as mentioned in the Supplementary Materials (Table S1).

### 2.5. Swelling and Degradation Study

Scaffolds composed of 8% alginate/6% gelatin were subjected to monitor the swelling or degradation studied over time in PBS and DMEM. Scaffolds in one set of experiments were covered with PBS, while the second set was covered with DMEM and incubated in a CO_2_ incubator at 37 °C. Daily, scaffolds were removed from the plate using forceps or a spatula and weighed and then placed back in the CO_2_ incubator at 37 °C. The experiment was discontinued when the scaffolds degraded.

### 2.6. Scanning Electron Microscopy

A scanning electron microscope (SEM) was used to confirm the presence of cells on the scaffolds’ surface. Four samples were taken for SEM: (a) 8% alginate/6% gelatin printed scaffold alone, (b) scaffold containing 7-day-cultured Fibroblast and HaCaT cells, (c) scaffold containing 14-day-cultured Fibroblast and HaCaT cells, and (d) scaffold containing 21-day-cultured Fibroblast and HaCaT cells. All of the studied scaffolds were fixed with cold 2.5% glutaraldehyde (2.5 h at 4 °C). Then, the scaffolds were washed with 0.1 M phosphate buffer twice to remove residual glutaraldehyde followed by the ethanol dehydration process. The dehydration was processed by applying increasing percentages of ethanol, starting with 30%, 50%, 70%, 80%, 90%, and 95%, and thrice with 100% ethanol. The incubation time of each scaffold was 30 min with each percentage of ethanol solution. The scaffolds were left to evaporate the remaining ethanol overnight. The SEM sample stubs were prepared by mounting carbon tape. The scaffolds were directly mounted with carbon tape. The samples were imaged using the Phenom pro Desktop imaging system, Nanoscience Instruments (USA).

### 2.7. Live/Dead Staining

For the live/dead fluorescence staining study, cells at the rate of 1 × 10^6^ cells per scaffolds (20 × 20 × 1 mm) were seeded for 7, 14, and 21 days. Every alternate day, the medium was replaced with fresh medium. After completion of the incubation period, scaffolds containing cells were stained using the Invitrogen live/dead imaging kit to measure cell viability. The dye mixture (20 μL) was added on each scaffold containing cells and incubated for 15 min. After incubation, images were captured, using the Invitrogen EVOS M5000 imaging system, from Thermo-fisher Scientific (USA), in which the green color indicates live cells and the red color indicates dead cells.

### 2.8. Cell Viability Assay

A 96-well plate was used for the MTT assay. Scaffolds were printed using 40 kPa, 6 mm/s, and 60% infill to obtain a gel sheet (30 × 30 × 1) mm that could be cut to fit the wells. A 7 mm diameter cutter was used to cut the disk and then placed into the wells. Then, the cells were seeded in a 96-well plate containing scaffolds and incubated for 1, 5, and 15 days. Complete DMEM was added 30 min after the settling of the cells on scaffolds. After completion of the incubation period, 20 μL of MTT solution was added to each well and incubated at 37 °C for 4 h. After 4 h, 150 μL of DMSO was added and incubated for another 15 min. The plate was then read at 570 nm using a the Biotek Synergy LX plate reader (USA).

### 2.9. Gene Expression Study

For the gene expression study, total RNA was extracted from the cells (Fibroblast and HaCaT) grown on a cell culture plate and scaffolds for 7 days. After completion of the incubation period, cells were collected and washed with PBS, and then 700 μL of trizol reagent was added. After vertexing for 3 min, 200 μL of chloroform was added, further vortexed for 10 min, and incubated at room temperature for 5 min. The supernatant was then collected in a new tube by centrifugation. Then, 300 μL of cold ethanol (70%) was added to the supernatant and thoroughly mixed. Then, cell lysate was transferred to an RNEasy spin column, and further steps were performed as per the instructions of the total RNA extraction kit, (Qiagen, USA). Nanodrop was used to quantify the RNA’s concentration and check its purity. A total of 250 ng of RNA concentration was used for the cDNA synthesis using the Invitrogen (USA) cDNA synthesis kit. cDNA was prepared by gently mixing the RNA sample with 1 µL of dNTP mix (10mM), 1 µL of Oligo(dT) primer, and 8 µL of nuclease-free water, followed by incubation at 65 °C for 5 min. After incubation, the reaction mixture was quickly transferred to an ice bath to cool for 2 min. Next, 4 µL of 5× first strand buffer, 2 µL of DTT (0.1 M), and 1 µL of RNAase out were added to the cooled mixture and incubated at 25 °C for 2 min. Finally, 1 µL of superscript II was added to the reaction mixture and incubated at 25 °C for 10 min. Then, incubation was continued at 42 °C for 50 min, followed by incubating at 70 °C for 15 min.

Further, gene expression was performed using QuantStudio 3 (Applied Biosystems, USA). GAPDH was taken as the reference control and the other studied genes, as mentioned in Table 1. The temperature condition was as follows: 95 °C as the initial temperature for 6 min, and then 95 °C for 15 s and 60 °C for 45 s for 40 cycles.

### 2.10. Melanin Content

For the melanin content analysis, Fibroblast cells were seeded on printed scaffolds for 1 week followed by seeding HaCaT cells on the same scaffold with Fibroblast cells. After another week, Melanocyte cells were seeded on the scaffolds grown with Fibroblast and HaCaT cells. After completion of the incubation, cells were extracted from the scaffold by using 104 mM of Nacl by gentle agitation for 2 min and centrifuge at 320× *g* for 2 min. As per the reported method [25], the cell pellet was further incubated with cell lysis buffer, and the lysate was centrifuged at 14,000 rpm for 10 min. The pellet was dissolved in 120 µL of 1M NaOH and incubated for 1 h. The standard was prepared by dissolving different concentrations of melanin in 1 NaOH and read at 476 nm.

## 3. Results

### 3.1. FTIR Analysis

**Figure 1:**
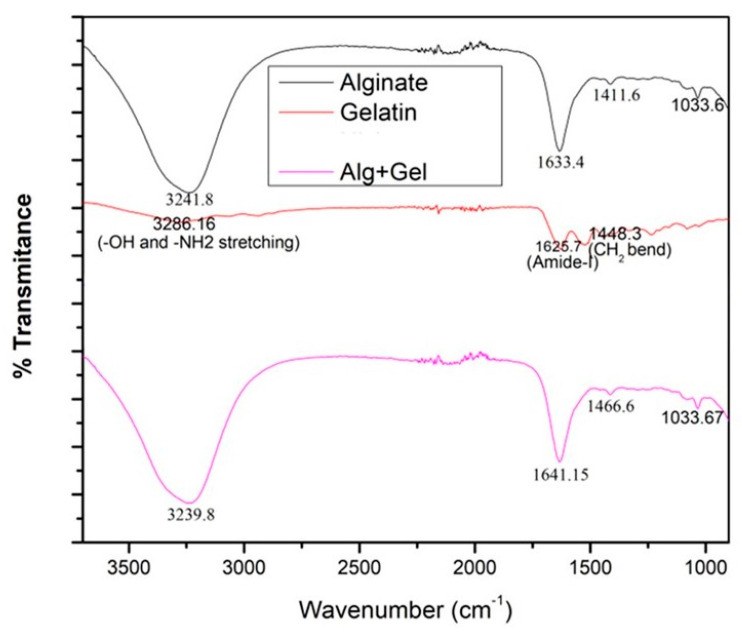
The above figure shows the FTIR results for gelatin, alginate, and the hydrogel, with characteristic peaks labeled.

The FTIR spectra graph (Figure 1) shows the three different materials analyzed by FTIR spectrophotometry to confirm the composition. The top line, black, shows the peaks corresponding to the functional groups of alginates at 3241 cm^−1^ and 1633 cm^−1^. The next line, red, shows the peaks corresponding to the functional groups of gelatins at 3266 cm^−1^ for CH2 and -OH stretching and 1625 cm^−1^ for amide stretching. The bottom pink line corresponds to 8% alginate/6% gelatin hydrogel. We found that peaks around 3239 cm^−1^ and 1641 cm^−1^ confirmed the presence of both gelatin and alginate in the gel.

### 3.2. Swelling and Degradation Study

**Figure 2:**
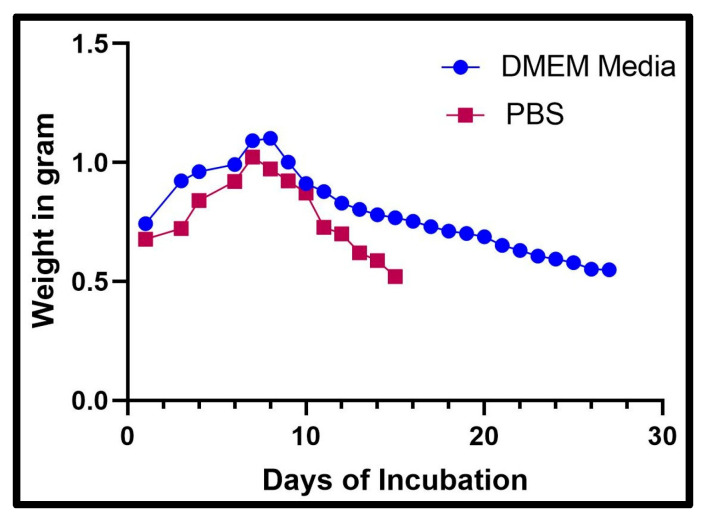
The graph above shows the recorded weight of a scaffold after being placed in DMEM and PBS each day.

The swelling and degradation experiments were concluded when the scaffold was too fragile to remove from the solutions. The data were graphed in terms of the weight of the scaffold in grams over days. The incubation period for the scaffold in DMEM (Figure 2) was 27 days, but the incubation period in PBS (Figure 2) was 15 days. There was a trend in both DMEM and PBS where the weight increased to 7 and 8 days and then decreased as degradation took place. The scaffolds in DMEM held their structure longer than the scaffolds in PBS, and the weight settled at about 0.549 g. The weight of the scaffold in PBS after 15 days was not recorded as the scaffold degraded. We found promising results where the scaffolds in DMEM retained their structure longer than the scaffolds in PBS. This result means that an 8% alginate/6% gelatin scaffold could be compatible with DMEM, which was used for the culture of cells, and the swelling results also demonstrated that gel was able to absorb media inside, which may help the cells to survive if cells grow inside the gel.

### 3.3. SEM Analysis

**Figure 3:**
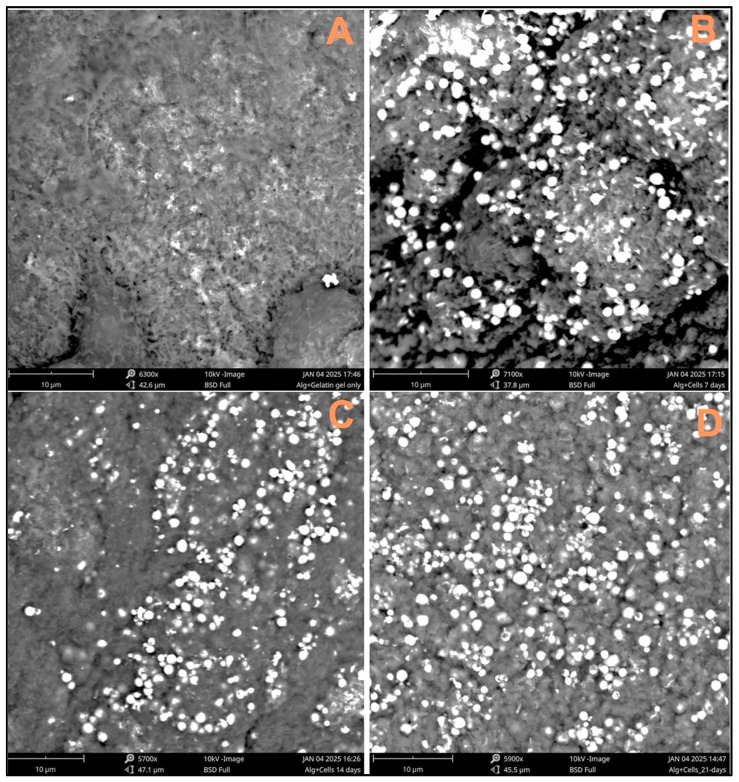
SEM images taken to confirm the presence of cells on the scaffold surface: (**A**) 8% alginate/6% gelatin printed scaffold alone, (**B**) scaffold containing 7-day-cultured Fibroblast and HaCaT cells, (**C**) scaffold containing 14-day-cultured Fibroblast and HaCaT cells, and (**D**) scaffold containing 21-day-cultured Fibroblast and HaCaT cells.

Three-dimensional-printed scaffolds with and without cells were evaluated for SEM analysis. The captured images indicate the cell cluster on the surface of the scaffolds, confirming the cells’ attachment and the spreading of the cells on the surface of the scaffolds. The maximum number of cells observed on the scaffolds was at 21 days (Figure 3D), as compared to the scaffolds with cells at 7 and 14 days.

### 3.4. Live/Dead Assay

**Figure 4:**
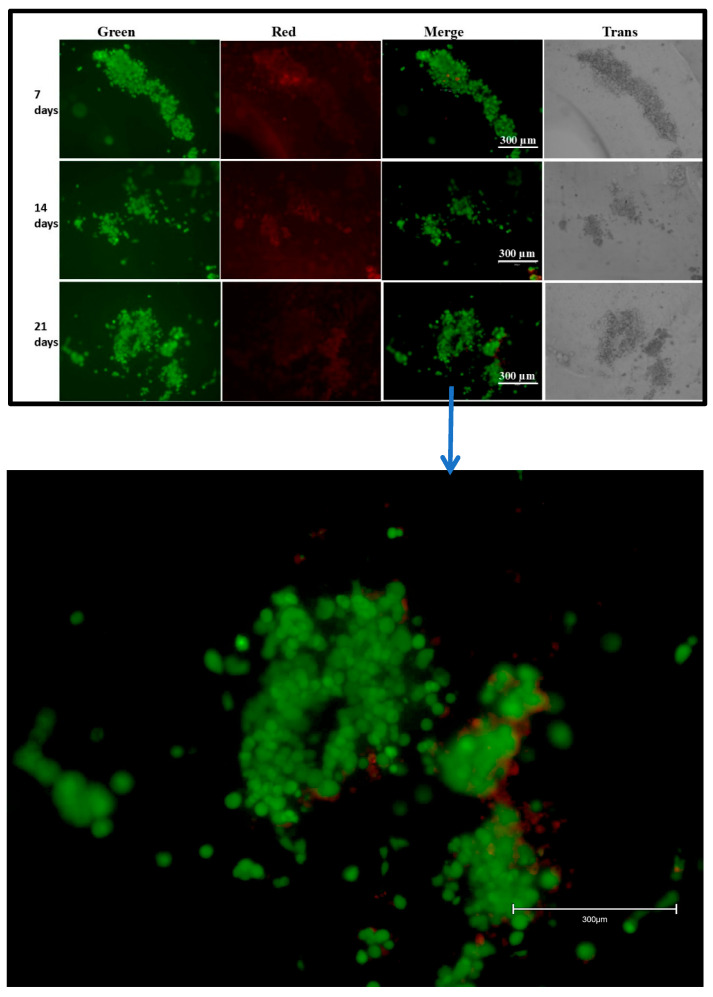
Live/dead assay for Fibroblasts/HaCaT cells grown on a scaffold, captured at 10× magnification. Combination of red, green, and trans images.

The live/dead assay was performed using a live/dead imaging kit to check the viability of the cells on the scaffolds after Fibroblast and HaCaT cells were cultured for 7, 14, and 21 days. Cell images were captured using a fluorescence microscope. As per the kit instructions, a green color is an indicator for viable cells, and a red color indicates dead cells. We observed more live cells, as indicated by the green color, on all our observed scaffolds (Figure 4). The results indicate that the 3D-printed alginate/gelatin scaffolds were compatible for cell growth and proliferation.

### 3.5. Cell Viability Assay

**Figure 5:**
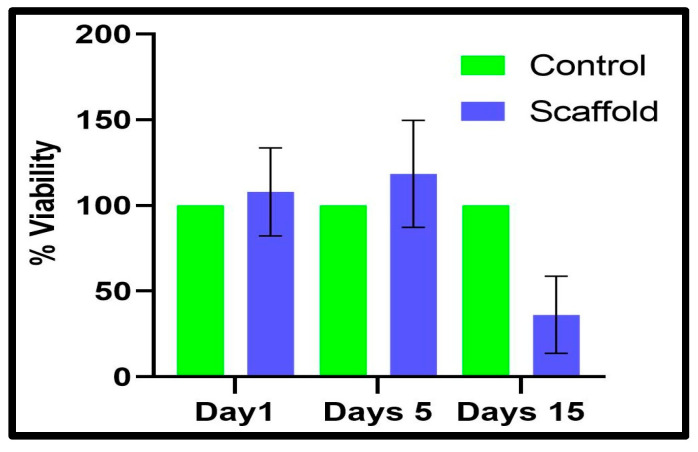
Percent viability was calculated by MTT assay for Fibroblast cells seeded on a scaffold compared to control cells grown on the plate.

The MTT assay was performed to determine the percentage of viable cells after cultured for 1, 5, and 15 days on scaffolds and in the cell culture plate. Three-dimensional-printed scaffolds were prepared for the MTT assay, as described in the Material and Methods Section 2.8. After the completion of the incubation period, both cells cultured in the well plate and on scaffolds were subjected to MTT dye treatment. Our finding suggested that cells cultured on scaffolds for 1 and 5 days showed similar viability compared to cells cultured in the well plate (Figure 5). However, decreased viability was found in the case of cells cultured on scaffolds for 15 days as compared to the cells cultured in the cell culture plate.

### 3.6. Gene Expression Study

**Figure 6:**
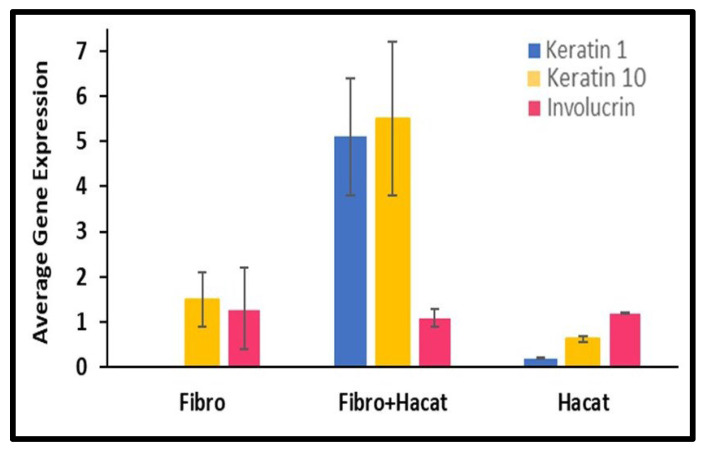
Average gene expression for Keratin 1, Keratin 10, and Involucrin as compared to the reference gene GAPDH. Fibroblast and HaCaT cells grown individually on a cell culture plate were used as a control for the Fibroblasts and HaCaT cells grown on a scaffold.

qPCR was performed to determine the gene expression of genes associated with skin tissue, as shown in Figure 6. The GAPDH gene was used as a reference control for Keratin 1, Keratin 10, and Involucrin. As shown in Figure 6, Keratin 10 was found to be present in the Fibroblasts and HaCaT cells grown both on scaffolds and the cell culture plate. Keratin 1 expression was negligible in Fibroblasts and less in HaCaT cells. Likewise, Keratin 10 and Involucrin expression was observed in both cells grown on scaffolds and cell culture plates. The Involucrin expression levels were stable in all of the studied groups. However, we observed an increase in the expression of Keratin 1 and Keratin 10 in co-cultured Fibroblast and HaCaT cells on scaffolds mimicking the conditions of skin layers.

### 3.7. Melanin Content in 2D and 3D Culture System

**Figure 7:**
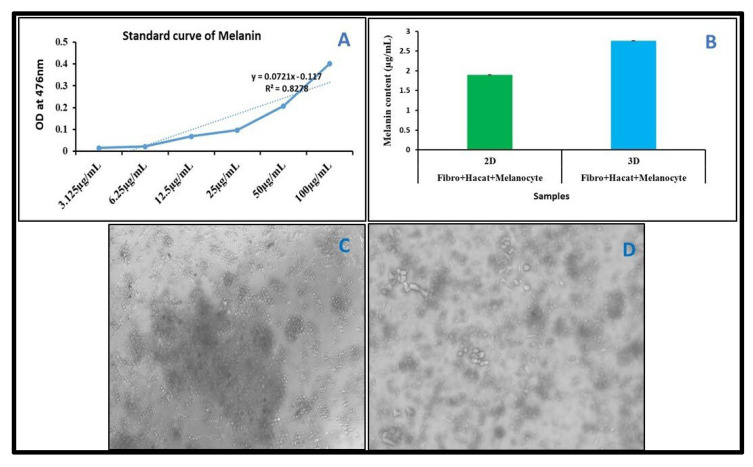
Melanin content quantification in 2D and 3D cultures. Cells were co-cultured in a cell culture plate and on a 3D scaffold for 21 days. (**A**) Standard curve for melanin content analysis. (**B**) Graph represents the melanin content in 2D and 3D cultures. (**C**) Images were captured after 21 days. Co-cultured cells in well plate (2D). (**D**) Images were captured after 21 days. Co-cultured cells on scaffold (3D).

For comparative melanin content analysis, Fibroblast, HaCaT, and Melanocyte cells were co-cultured, as described in the Materials and Methods Section. After the completion of the incubation period, cells were collected and lysed, and the melanin content was quantified using a spectrophotometer in both 2D- and 3D-cultured cells. Our melanin content analysis results showed that melanin production in 3D co-cultured cells was higher compared to co-cultured cells in 2D (Figure 7B).

## 4. Discussion

The ideal hydrogels for tissue engineering should allow nutrients and chemicals to diffuse, mimic natural tissues as closely as possible, and create an environment that supports cell proliferation. Hydrogels must contain collagen or collagen derivatives (like gelatin) to be effective at tissue regeneration since a large amount of collagen is present in the extracellular matrix (ECM) [26]. Additionally, scaffolds for tissue engineering are frequently constructed using alginate, a naturally occurring polymer. Alginate is essential for creating hydrogel mixtures for 3D printing because it can make the ink more viscous, which allows the solution to be extruded, and it can crosslink with divalent salts in mild conditions [27]. Alginate does, however, have certain drawbacks, including poor cellular motility and adhesion [28]. Gelatin–alginate mixtures are now frequently used to print scaffolds for tissue engineering. However, these dual mixes’ mechanical strength is typically insufficient to print scaffolds with larger diameters, and the addition of solid support elements like carbon nanotubes and bioactive glass particles frequently increases their endurance [27,28,29]. For 3D hydrogel constructs to remain stable, crosslinking procedures are necessary [30]. Ionic interactions between Ca^2+^ ions and the carboxyl groups of the guluronic acid residues of the two adjacent alginate chains were the primary cause of the physical crosslinking of hydrogels by CaCl_2_ treatment. Additionally, Ca^2+^’s chelating center coordinating role stabilizes these connections [31]. Likewise, Ca^2+^ ions influence the structure of the gelatin network by interacting with the carboxylic acid groups on the gelatin polypeptides [32]. The FTIR examination verified the scaffold’s composition, the presence of the gelatin crosslinking reaction, and the alginate crosslinking process via Ca^2+^ ions. The -C=O stretching band (amide-I) and the -NH bending vibration (amide-II) for immaculate gelatin were observed at ~1625 and ~1446 cm^−1^, respectively. Additionally, -CN stretching vibrations are responsible for the bands at ~1334 and ~1238 cm^−1^, whereas -CH stretching and bending vibrations are responsible for the bands at ~2942 and ~1446 cm^−1^, respectively [33,34]. For clean sodium alginate, it is possible to clearly see the main characteristic bands associated with the carbonyl group’s -OH stretching (broadband approximately 3241 cm^−1^), asymmetric stretching vibration (1633 cm^−1^), and symmetric stretching vibration (1411 cm^−1^) [35]. The shift to 1641 and 1466 cm^−1^ in the band associated with COO stretching indicates that this group is associated with Ca^2+^ during alginate crosslinking [36]. The swelling and degradation study findings showed that the scaffolds’ weights increased throughout the first seven days in both DMEM and PBS (pH 7.4). Later, it was found that the scaffolds’ weights decreased in both DMEM and PBS (pH 7.4). As per the previous report, hydrogel showed similar behavior after being incubated with PBS solution [37,38]. Our SEM analysis confirmed the attachment and spreading of cells, even at 21 days on 3D scaffolds’ surfaces. Another group also reported cells’ attachment and spreading on the surface of alginate gel using SEM [39]. Furthermore, the live/dead assay showed viable cells even at 21 days on the scaffolds. A similar study was performed by another group using an alginate/gelatin scaffold with a different composition using different cells type and reported the viability of the cells on the scaffold using the live/dead assay [40,41]. Further, our cell viability results on the 8% alginate/6% gelatin 3D-printed scaffold from the MTT assay showed the comparably better viability in the case of culturing for 1 and 5 days; however, decreased viability was observed in the case of 15 days on the scaffolds compared to cells grown on the cell culture plate. Since previous research has shown that alginate alone cannot sustain mammalian cell attachment and proliferation due to a lack of arginine–glycine–aspartate (RGD) tripeptide, the loss in viability may be related to the release of gelatin from the Alg-Gel scaffold [26,42].

In another study on C2C12 muscle cells using an 8% alginate/6% gelatin 3D-printed scaffold, the maximum viability of the cells was reported at 7 days by the MTT assay [43]. In another study, the CaCl_2_-EDC- and EDC–CaCl_2_-induced scaffolds were compared [44], which showed that the viability of the HSF cells on the EDC–CaCl_2_ scaffold increased more significantly after 7 days of the cell culture. This finding suggests that EDC–CaCl_2_ scaffolds are better suited for the proliferation of HSF cells than CaCl_2_–EDC under the same conditions [44]. Another group prepared a scaffold with 5% alginate and 6% gelatin and cultured aortic root sinus smooth muscle cells (SMCs) and aortic valve leaflet interstitial cells (VICs) for 7 days; they reported around 90% cell viability [16]. Our viability results on the scaffolds showed better viability as compared to a study conducted previously on a different scaffold. From the qPCR results, we found a high expression of Keratin 1 and Keratin 10 genes when Fibroblasts and HaCaT cells were co-cultured on a scaffold mimicking the conditions of skin layers. For skin equivalents, another research group created innovative 3D-printed cell culture inserts. They co-cultured Fibroblast and Keratinocyte cells in an air–liquid interface, assessed the expression of Keratin 10 using immunofluorescence, and reported high Keratin 10 expression [45]. Further, the co-culture study was performed using three different skin cells, i.e., Fibroblasts, HaCaT, and Melanocytes, in both cell culture plates and on 8% alginate/6% gelatin 3D-printed scaffolds. We found the maximum melanin content to be in the co-culture on the 3D scaffold as compared to the cell co-cultured in the cell culture plate. It has previously been reported that in 3D culture systems, cell experiences enhanced cell–cell and cell–matrix interactions as compared to 2D culture, leading to improved cell morphology, viability, and differentiation. The process of melanogenesis, or the synthesis of melanin, depends on these interactions. Melanocytes use a complex signaling network to communicate with nearby Keratinocytes in the epidermis. These interactions are maintained or even improved in 3D models, which results in more effective melanin transport and production [46,47,48]. In another co-culture study on melanin synthesis under skin-like conditions and the impact of UV light, the research group performed co-cultured Keratinocytes and Melanocytes for three days in DMEM/Ham’s F12 (3:1), and then the same was co-cultured in an air–liquid interface for eleven days [49]; they found melanin in both Melanocytes and the surrounding Keratinocytes cells [49]. In another study, Melanocyte–Keratinocyte were co-cultured for 14 days on 3D collagen gel containing FGF-2 and FGF-7 polypeptides [50]. It was found that the Melanocytes reside at the base, whereas a significant quantity of melanin was also seen in the lower layer [50].

There are a lot of studies on alginate–gelatin gel, but most of these studies focus on physical properties like viscosity, rheology, printing, pore size, and stability, based on the percentage and ratio of alginate–gelatin. There are few reports of alginate–gelatin scaffolds in biological applications in tissue engineering, with most research focused on bone tissue engineering, but current studies focus on applications in wound healing as we used all three skin cell lines. Our unique finding in terms of longtime viability and increased melanin production can boost the research focus on the application of alginate/gelatin scaffolds in wound healing.

## 5. Conclusions

A single biomaterial cannot meet all the criteria needed for producing functional 3D-printed scaffolds. This work successfully generated a hydrogel scaffold using an alginate–gelatin blend for extrusion-based 3D printing. Our finding suggested that an alginate and gelatin scaffold can be used as a cellular matrix for epithelial cell culture. Gel composed of 8% alginate/6% gelatin is ideal since it can be easily 3D-printed in any number of shapes, and the cells can be incorporated or seeded. This degradation study indicated that the alginate/gelatin scaffold would be a viable option for cell culture in DMEM due to its ability to retain its structure for more than 25 days. Overall, the studied scaffold is superior to existing ones in terms of printability and cell viability/biocompatibility, and our scaffolds provide a suitable 3D environment for cells to interact with each other to produce melanin. Further, in vivo and ex vivo experiments are needed to validate the results for future applications in tissue engineering for wound healing.

## Figures and Tables

**Table 1:** Primer details used for qPCR analysis.

S. No.	Gene Name	Primer Sequence	Size (bp)
**1.**	Keratin 1	Forward:5′-ATTTCTGAGCTGAATCGTGTGATC-3′ Reverse:5′-CTTGGCATCCTTGAGGCAATT—3′	24 22
**2.**	Keratin 10	Forward:5′-GTAGTCAGTTCCTTGCTCTTTCA-3′ Reverse:5′-TGATGTGAATGTGGAAATGAATGC-3′	22 24
**3.**	Involucrin	Forward:5′-GGGTGTTATTATGTTGGGTGG-3′ Reverse:5′-GCCAGGCCAAGACATTCAAC-3′	21 20
**4.**	GAPDH	Forward:5′-TGCACCACCAACTGCTTAGC-3′ Reverse:5′-GGCATGGCTGTGGTCATGAG-3′	20 20

## Data Availability

All data connected to this study are available from the authors on request.

## Supplementary Materials

The following supporting information can be downloaded at: https://www.mdpi.com/article/10.3390/bioengineering12030270/s1, Table S1: The following scaffolds were printed using alginate/gelatin gel for this study. Figure S1: Images A and B show the scaffold immediately after printing. Images C and D show the scaffold after CaCl_2_ was applied and polymerization occurred.
