# Supplementary material for: Three-Dimensional Printing of Hydrogel as Skin Substitute and Comparative Evaluation of Melanin Production
Source: Bioengineering (Basel). 2025 Mar 9;12(3):270. doi: 10.3390/bioengineering12030270 (PMC11939583; doi:10.3390/bioengineering12030270)
Supplement: Supplementary file 1 [file bioengineering-12-00270-s001.zip › bioengineering-3477454-supplementary.pdf]

Table 1. The following scaffolds were printed using 8% alginate/6% gelatin gel for study.

Scaffold	Alg (%)	Gel (%)	Dimension (mm)	Pressure (Kpa)	Infill (%)	Speed mm/s	Pattern
1	8	6	20X20X3	40	20	6	Grid
2	8	6	20X20X3	40	20	6	Grid
3	8	6	20X20X3	40	20	6	Grid
4	8	6	20X20X3	40	20	6	Grid
5	8	6	20X20X3	40	20	6	Grid
6	8	6	30X30X3	40	20	6	Grid
7	8	6	30X30X3	40	20	6	Grid
8	8	6	30X30X3	40	20	6	Grid

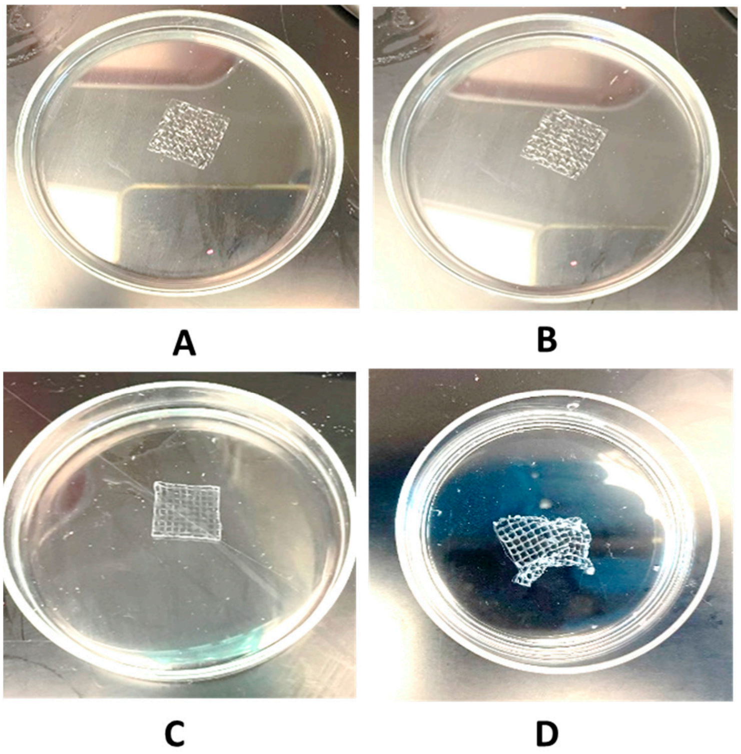

Figure1. The images A and B, scaffold immediately after printing. The images C and D scaffold after $CaCl_2$ applied and polymerization occurred.
